# AI for psychiatric training and education

**DOI:** 10.1192/j.eurpsy.2025.241

**Published:** 2025-08-26

**Authors:** D. Eraslan, P. Thomann

**Affiliations:** Psychiatry, SRH Klinikum Karlsbad Langensteinbach, Karlsbad, Germany

## Abstract

**Abstract:**

Artificial intelligence (AI) is transforming psychiatric training and education by enhancing diagnostic accuracy, improving therapeutic decision-making, and personalizing learning experiences for trainees. AI-driven simulations, virtual patients, and natural language processing (NLP)-based assessments allow for more effective skill development in psychiatric diagnosis and psychotherapy. Machine learning models provide evidence-based guidance, reinforcing clinical reasoning and treatment strategies. Ethical considerations, including patient confidentiality and bias mitigation, remain central to AI implementation in training. This session explores the latest advancements in AI-driven psychiatric education, discussing practical applications, challenges, and future directions for integrating AI into clinical training programs.

**Keywords:**

AI, psychiatry, education, machine learning, clinical training

**References:**

1. Ahmed, M., & Rush, A. J. (2023). Artificial intelligence in psychiatry: Current applications and future directions. *Journal of Psychiatric Research*, 157, 106-121.

2. Ryu, S., & Kim, H. (2022). AI-based learning tools for medical education: A systematic review. *Medical Teacher*, 44(5), 512-520.

3. Luxton, D. D. (2021). Ethical implications of AI in mental health care. *Journal of Ethics in Mental Health*, 12, 1-14.

**Disclosure of Interest:**

None Declared

